# Supplementary material for: Rating pome fruit quality traits using deep learning and image processing
Source: Plant Direct. 2024 Oct 8;8(10):e70005. doi: 10.1002/pld3.70005 (PMC11461139; doi:10.1002/pld3.70005)
Supplement: Supplementary file 1 — Supplemental Method Figure S1. Starch analysis images thresholded with (a) and without (c) a division line, and identified ROIs of the above threshold images with (b) and without (d) a division line. Without a division line, one incorrect ROI was identified (b), while with a division line (d) two correct ROIs were identified. Iodine‐stained regions were correctly identified in thresholds with a division line (c, d). Supplementla Method Figure S2. Average percent starch rating of the limited rating experience rating (blue, N = 18), novice rating experience (yellow, N = 9), professional rating experience (orange, N = 9), and macro starch rating output (black). Images 1–36 iodine‐stained ‘Granny Smith cross‐sections. Images 37–72 iodine‐stained ‘Gem’ pear cross‐sections. Supplemental Method Figure S3. Average percent starch rating of all participants of the visual starch rating assessment (black, N = 36) compared to macro starch rating output (orange). Images 1–36 iodine‐stained ‘Granny Smith’ apple cross‐sections. Images 37–72 iodine‐stained ‘Gem’ pear cross‐sections. Cross‐sections. [file PLD3-8-e70005-s002.pdf]

Supplemental Method - Starch Rating

The code of the ImageJ macro for starch rating is composed of six key regions: (1) a "fresh start" region where appropriate settings are applied and fields are reset for proper operation of the macro, (2) code which allows the user to choose input and output directories, (3) enabling of batch mode to fetch all images found in the input folder, (4) calculation of starch present in cross-sections, (5) calculation of total cross section area, and (6) saving data as a comma-separated file (CSV) to the output folder, where percent starch content is calculated per image.

When first initialized, appropriate settings are first applied to ImageJ ensuring accurate measurement of starch content. This includes (1) closing of any windows open in ImageJ, (2) resetting of the Region of Interest Manager (roiManager), (3) clearing data from the log file and subsequently closing the window, (4) setting the option "Black Background" to true, (5) enabling the display of file names for measurements, (6) setting the foreground color to white and background color to black, and (7) setting the width of drawn lines to one pixel. These settings must be changed for processes of the macro to run correctly.

Input and output directories are next designated by the user. The input folder should contain isolated cross-section images stained with iodine saved as a JPEG (.jpg), PNG (.png), or TIFF (.tif) format, with no limit on the number of images stored in the folder; all images of the input folder will be processed by the macro. The output folder is where threshold images of (a) starch area, (b) total cross-section area, and (c) the final comma-separated "Results.csv" file is saved. Threshold images of starch area and cross-section area are saved as a JPEG with the line "starch.jpg" or "totalarea.jpg" respectively added to the end of the original file name. After the input and output directories are chosen by the user, the macro will create a log in ImageJ with column titles which will be present in the final results file in comma-delimited format: data is added to this log following image analysis.

Batch mode is then enabled by the macro to fetch and process all images found in the input folder. Following this step all code is applied on a per-image basis; the script will repeat code until all images of the input folder have been processed. The macro will repeat code on subsequent images in the input folder until the last image is processed, where the log file is saved as "Results.csv" in the output folder.

Depending on the number of ROIs, the area of each region is identified by ImageJ as an alphabetical variable (C-L), which are later used by the macro to calculate starch content. The script is currently limited to ten identifiable ROIs, though could be modified to incorporate a higher number of regions. Based on our data set with apple and pear, ImageJ identifying more than six ROIs is uncommon.

Due to ImageJ's region of interest (ROI) identification process, a thin line must be drawn by the macro to divide the image into two halves. If this step does not occur any images where starch runs the full circumference of the cross-section (similar in shape to a donut) the area calculated

by ImageJ will be that of the inner non-iodine-stained area (Figure 6). Otherwise ImageJ identifies the inner region of such cross-sections as the primary region-of-interest (ROI), instead of the actual iodine-stained region. With the image divided into two halves, the iodine-stained area of the image is correctly identified since no single ROI can run the full circumference of the cross-section.

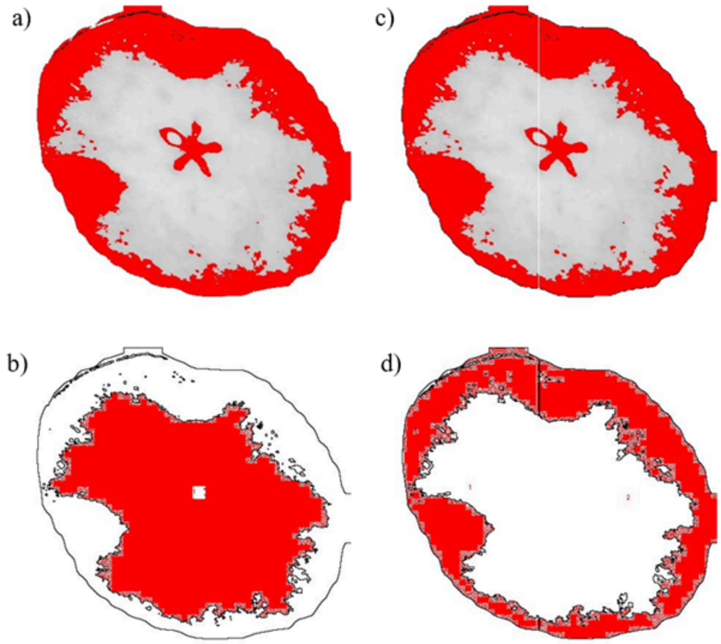

Figure S1. Starch analysis images thresholded with (a) and without (c) a division line, and identified ROIs of the above threshold images with (b) and without (d) a division line. Without a division line one incorrect ROI was identified (b), while with a division line (d) two correct ROIs were identified. Iodine-stained regions were correctly identified in thresholds with a division line (c, d).

A starch threshold image is saved to the output folder as a JPEG with “starch.jpg” appended to the image’s original file name. The macro will then pause for 100 milliseconds permitting file saving on slower systems. After another 100 millisecond pause, a second threshold is then applied to the image to calculate the total area of the cross-section. Using the “Analyze Particles” function, the total area of the cross-section threshold is calculated, and the threshold is saved to the output folder with “total_area.jpg” appended to the image’s original file name. The total area of the cross section is saved by ImageJ as variable “B”.

The code then assesses the number of ROIs, calculates the sum ROI area, and divides the sum ROI area by the total cross-section area to calculate percent starch area of the cross-section, saving this data to the macro’s log file. Using percent starch content best-fit polynomial lines of established rating scales, the starch rating for each image is also calculated and rounded to the nearest integer by the macro. Established rating scales calculated include: the Cornell Chart (Blanpied and Silsby 1992), Jonagold (REF), Purdue (REF), and University of California - Davis’

‘Granny Smith’ starch rating scales (Mitcham et al 1996). These values are found in the designated output folder “Results.csv” file.

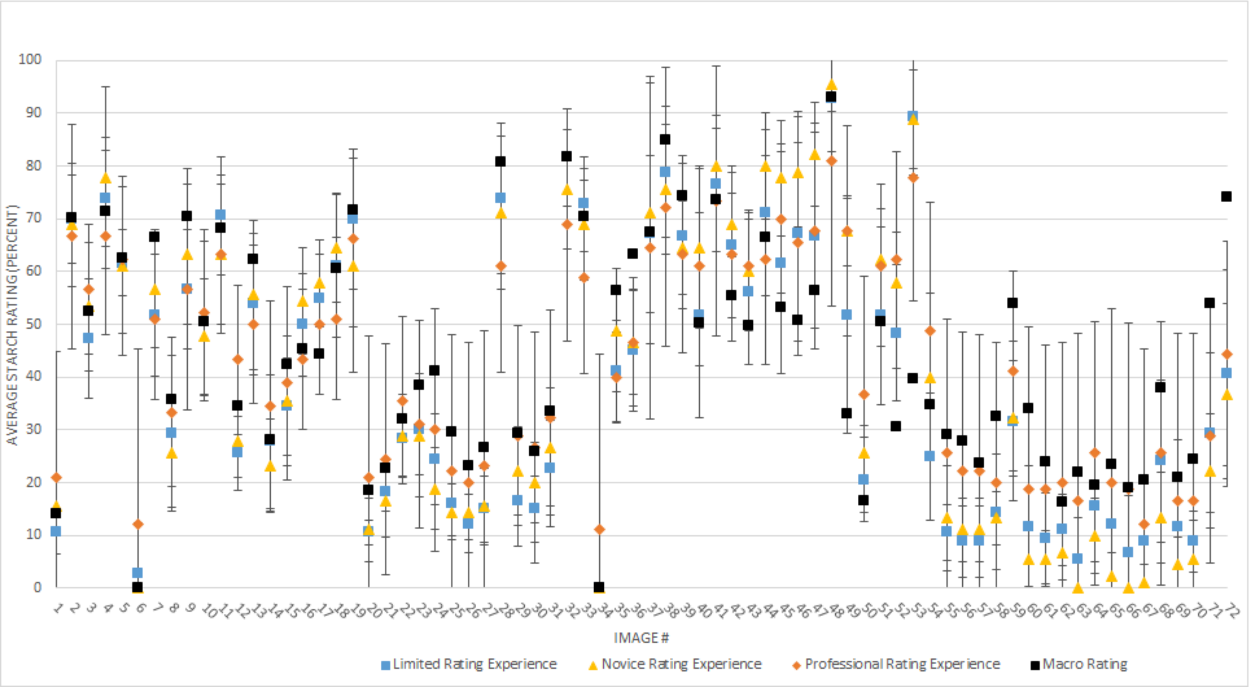

Figure S2. Average percent starch rating of the limited rating experience rating (blue, N = 18), novice rating experience (yellow, N = 9), professional rating experience (orange, N = 9), and macro starch rating output (black). Images 1-36 iodine-stained ‘Granny Smith cross-sections. Images 37-72 iodine-stained ‘Gem’ pear cross-sections. (https://drive.google.com/drive/u/0/folders/1PD_WQrnZfrZYaZVfgNmpeVp0a_Bdx5o5)

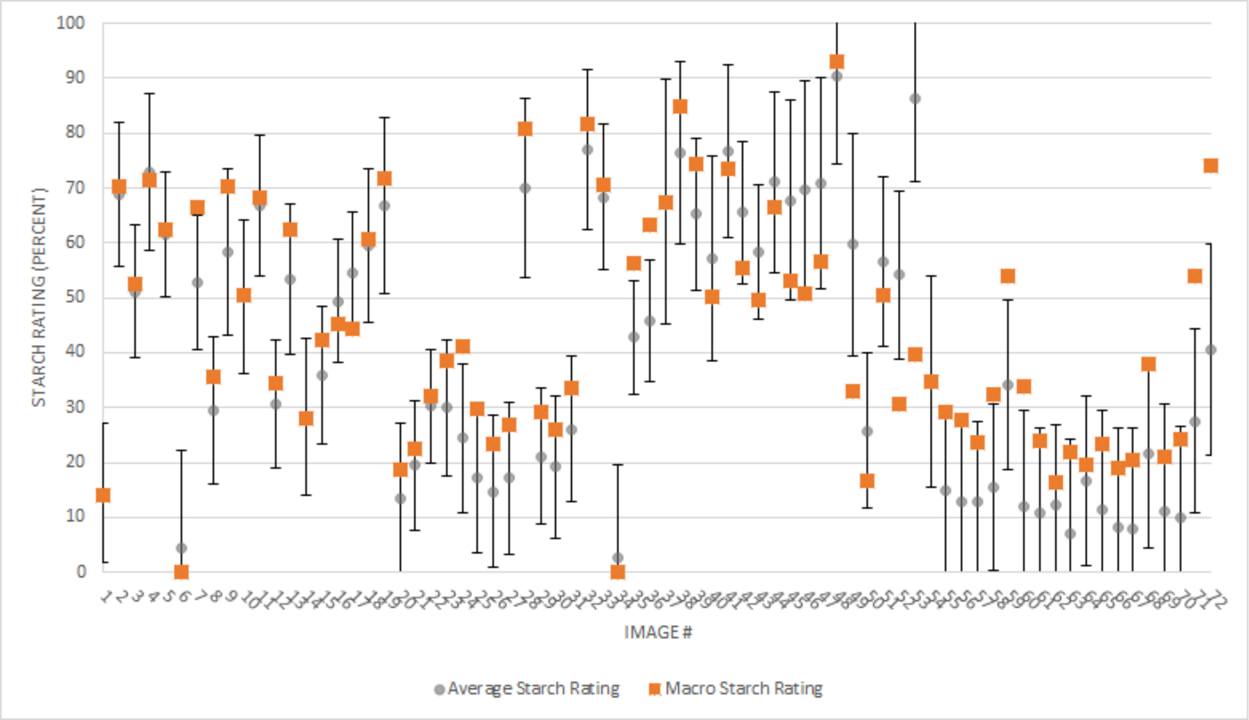

Figure S3. Average percent starch rating of all participants of the visual starch rating assessment (black, N = 36) compared to macro starch rating output (orange). Images 1-36 iodine-stained ‘Granny Smith’ apple cross-sections. Images 37-72 iodine-stained ‘Gem’ pear cross-sections.
(https://drive.google.com/drive/u/0/folders/1PD_WQrnZfrZYaZVfgNmpeVp0a_Bdx5o5)
