# Supplementary material for: Rating pome fruit quality traits using deep learning and image processing
Source: Plant Direct. 2024 Oct 8;8(10):e70005. doi: 10.1002/pld3.70005 (PMC11461139; doi:10.1002/pld3.70005)
Supplement: Supplementary file 2 — Figure S9. Starch content estimation from Granny and human participants with different levels of skills. (A), (C), (E) are score comparisons for apple cross‐sections, while (B), (D), (F) are for pears. Participant skill levels are limited in (A) and (B), novice in (C) and (D), and experienced in (E) and (F). (G) and (H) are residual plots for all the apple and pear data, respectively. 36 apples from a variety of cultivars and 36 ‘Gem’ pears were evaluated. Numbers of participants in each skill group are as follows: 18 participants have limited experience, 9 consider themselves novice technicians, and 9 are experts. LMS: Least Mean Square; RMSE: Root Mean Squared Error. PCC: Pearson Correlation Coefficient. Figure S10. Examples of pears rated as .5 or 1 by technicians are shown in (A) and (B); examples of pears rated as 1 by Granny are shown in (C). (A) and (B): The top row contains images of the full try where the pears in question were extracted from. Pear are numbered from 1–18, starting from the top right corner. Second and third rows are extracted pears from the corresponding tray, their location on the tray, and Granny's color rating. Figure S11. Pear blush rating from technicians compared to blush estimation from Granny. Granny predicted blush scores (y‐axis) are compared to (A) the average rating from three technicians, (B) ratings from technician 1, (C) ratings from technician 2, and (D) ratings from technician 3. LMS: Least Mean Square; RMSE: Root Mean Squared Error. PCC: Pearson Correlation Coefficient. (E) and (F) are examples of the four pear images with the highest standard deviations from the three technician ratings. [file PLD3-8-e70005-s001.pdf]

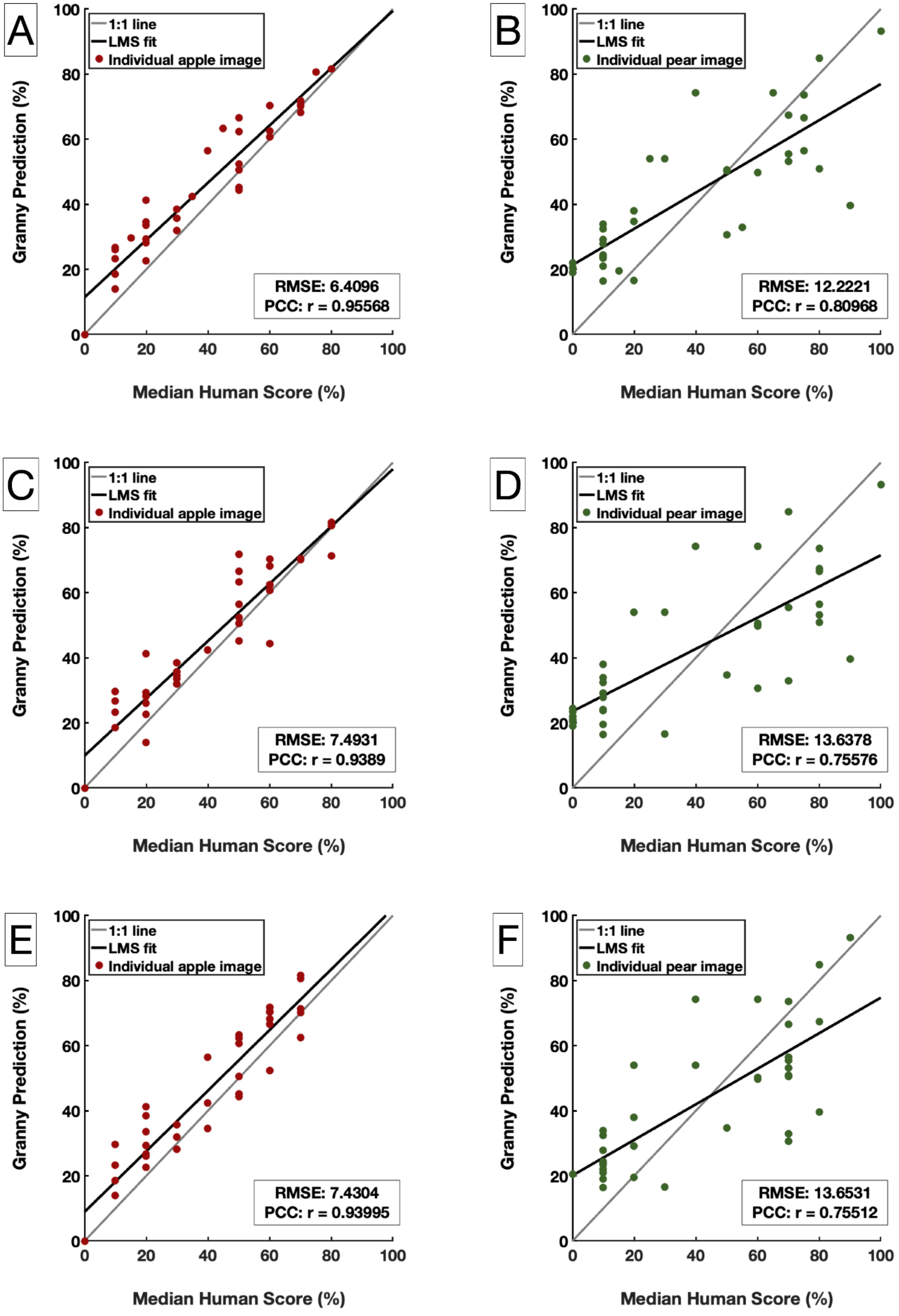
A
1:1 line
LMS fit
Individual apple image
RMSE: 6.4096
PCC: r = 0.95568
Granny Prediction (%)
Median Human Score (%)
B
1:1 line
LMS fit
Individual pear image
RMSE: 12.2221
PCC: r = 0.80968
Granny Prediction (%)
Median Human Score (%)
C
1:1 line
LMS fit
Individual apple image
RMSE: 7.4931
PCC: r = 0.9389
Granny Prediction (%)
Median Human Score (%)
D
1:1 line
LMS fit
Individual pear image
RMSE: 13.6378
PCC: r = 0.75576
Granny Prediction (%)
Median Human Score (%)
E
1:1 line
LMS fit
Individual apple image
RMSE: 7.4304
PCC: r = 0.93995
Granny Prediction (%)
Median Human Score (%)
F
1:1 line
LMS fit
Individual pear image
RMSE: 13.6531
PCC: r = 0.75512
Granny Prediction (%)
Median Human Score (%)

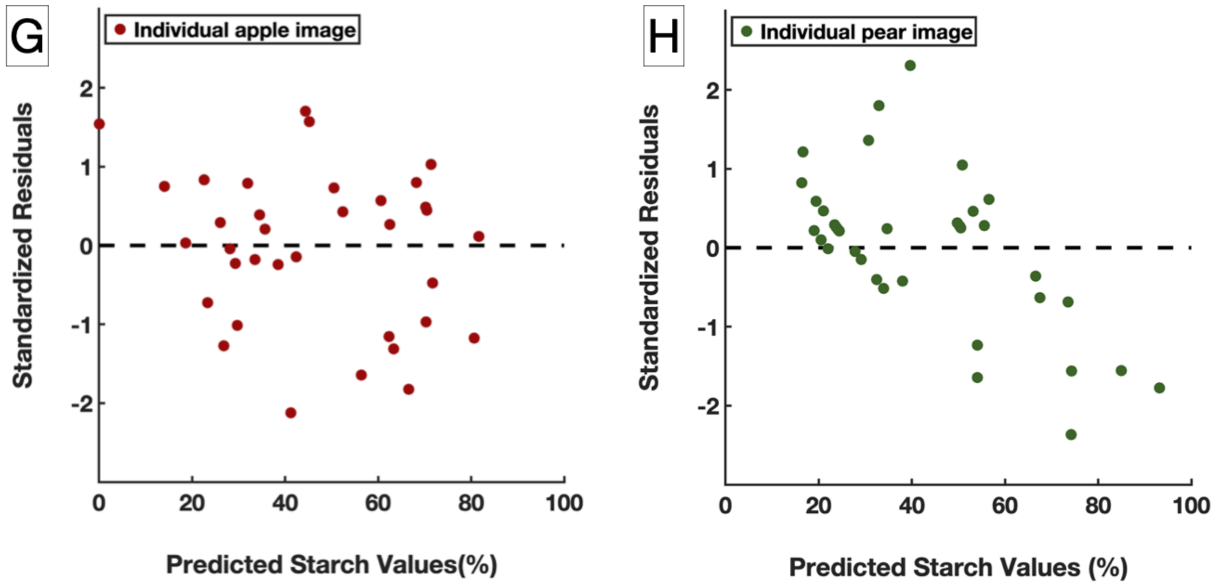

Supplemental Figure 9. Starch content estimation from Granny and human participants with different levels of skills. (A), (C), (E) are score comparisons for apple cross-sections, while (B), (D), (F) are for pears. Participant skill levels are limited in (A) and (B), novice in (C) and (D), and experienced in (E) and (F). (G) and (H) are residual plots for all the apple and pear data, respectively. 36 apples from a variety of cultivars and 36 'Gem' pears were evaluated. Numbers of participants in each skill group are: 18 participants have limited experience, 9 consider themselves novice technicians, and 9 are experts. LMS: Least Mean Square; RMSE: Root Mean Squared Error. PCC: Pearson Correlation Coefficient.

A. Pear rated as 0.5 by technician:

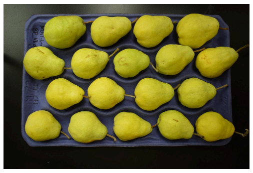	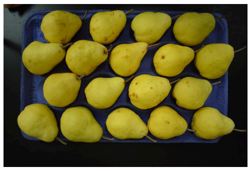	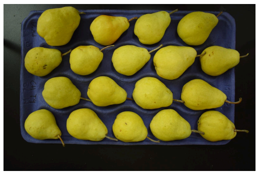	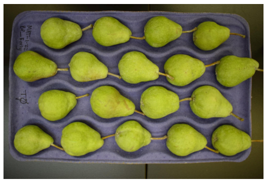
Pear No.: 6<br>Granny: 4.5<br>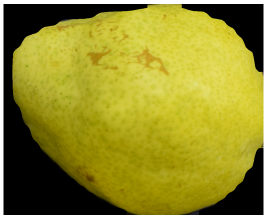	Pear No.: 15<br>Granny: 4.5<br>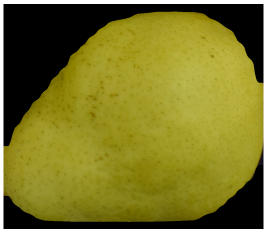	Pear No.: 9<br>Granny: 5<br>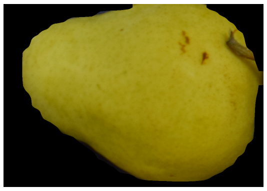	Pear No.: 1<br>Granny: 2.5<br>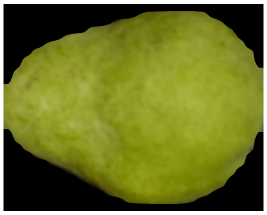
			Pear No.: 16<br>Granny: 2.5<br>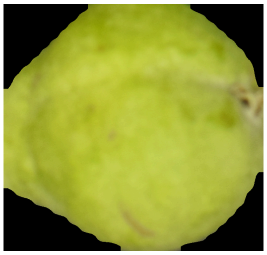

B. Pear rated as 1.0 by technician:

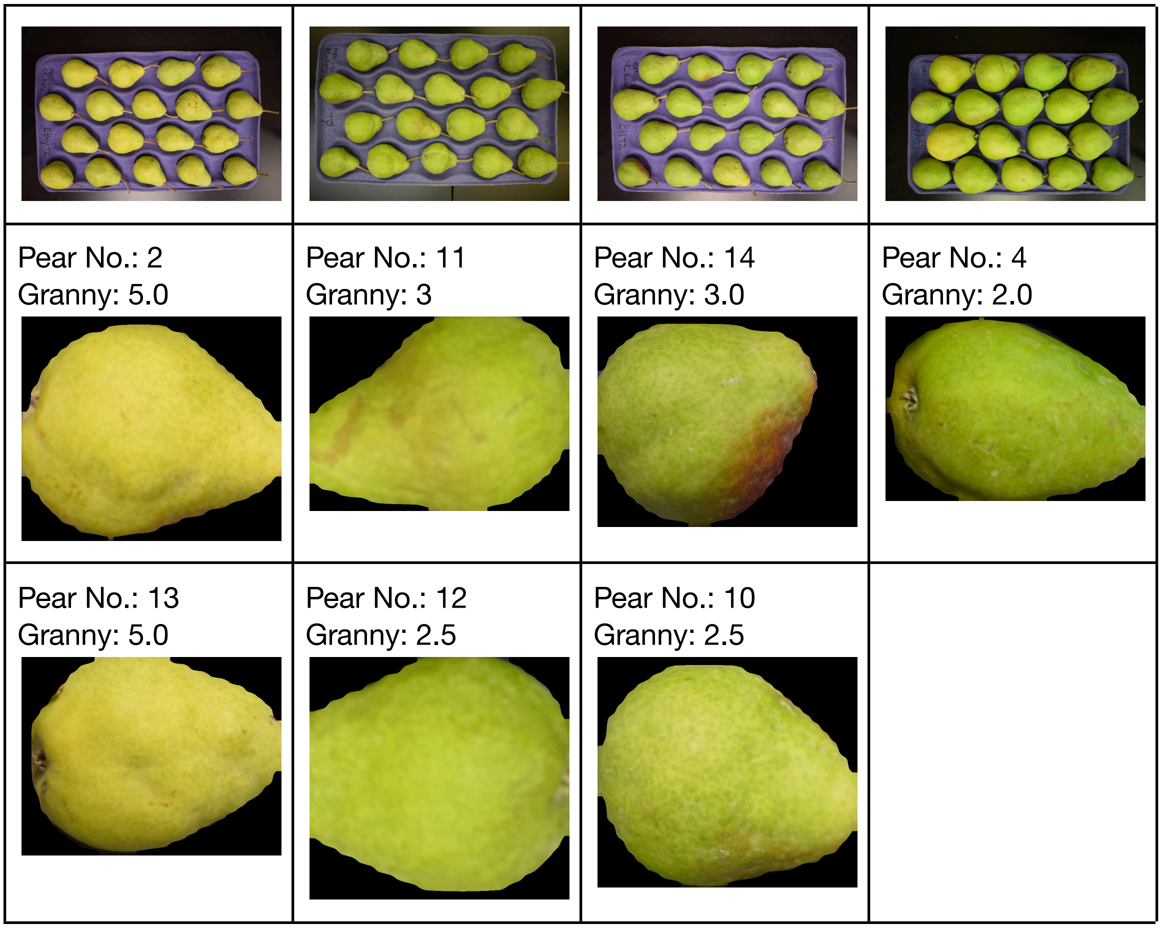

C. Examples of Granny's predictions for 1.0 groups:

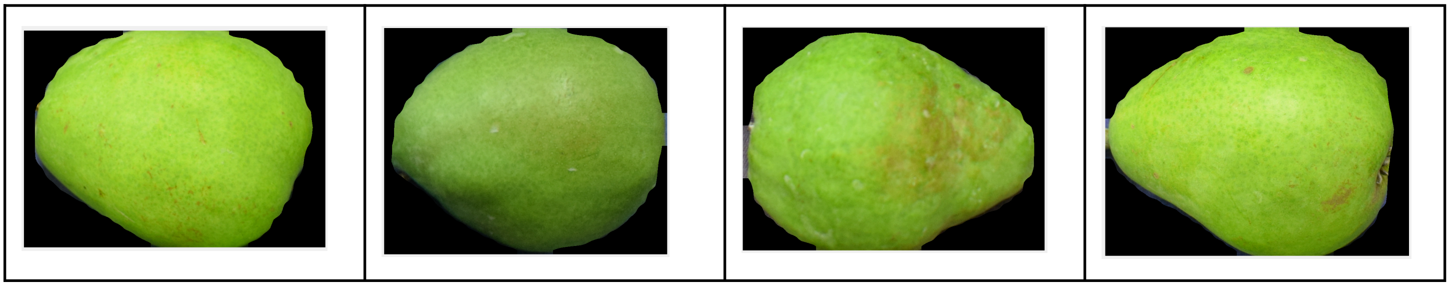

Supplemental Figure 10. Examples of pears rated as 0.5 or 1 by technicians are shown in **(A)** and **(B);** examples of pears rated as 1 by Granny are shown in **(C)**. **(A)** and **(B)**: The top row contains images of the full try where the pears in question were extracted from. Pear are numbered from 1-18, starting from the top right corner. Second and third row are extracted pears from the corresponding tray, their location on the tray, and Granny's color rating.

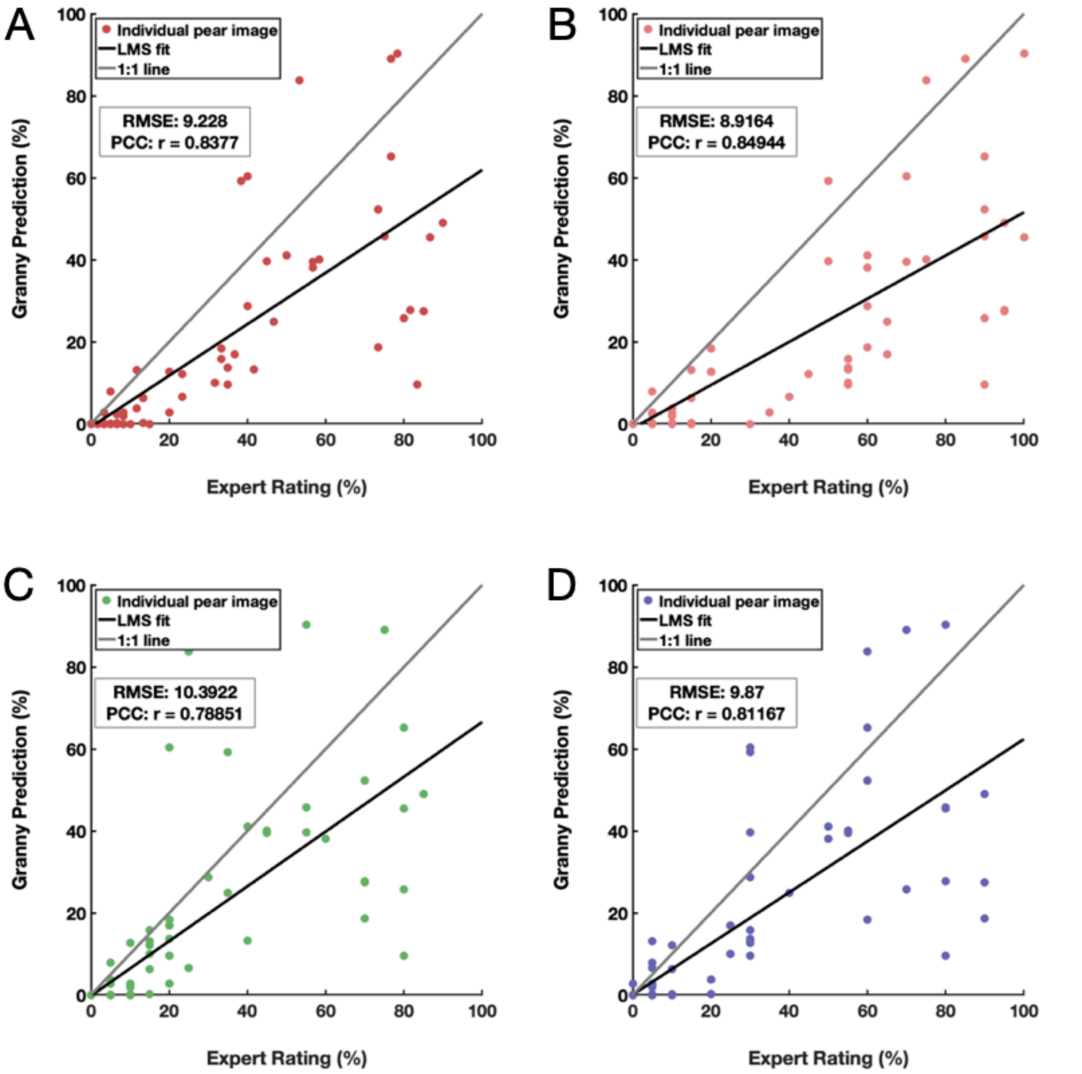

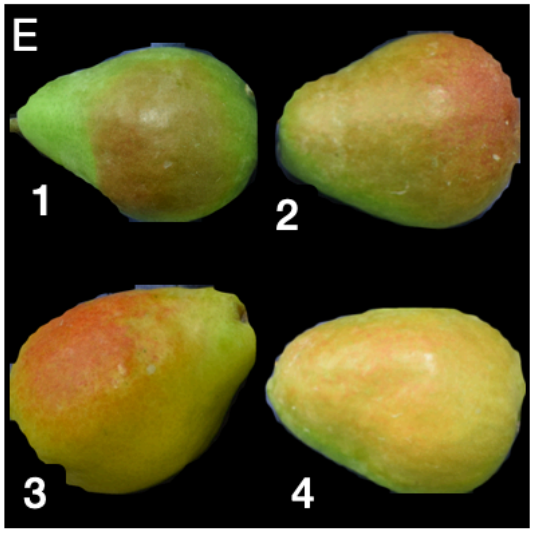

F	Pear1	Pear2	Pear3	Pear4
Granny	60.35	83.82	16.98	18.42
Tech1	70	75	65	20
Tech2	20	25	20	20
Tech3	30	60	25	60
STDEV*	26.46	25.66	24.66	23.09

STDEV: Standard deviation calculated from the three technician ratings.

Supplemental Figure 11. Pear blush rating from technicians compared to blush estimation from Granny. Granny predicted blush scores (y-axis) are compared to (A) the average rating from three technicians, (B) ratings from technician 1, (C) ratings from technician 2, and (D) ratings from technician 3. LMS: Least Mean Square; RMSE: Root Mean Squared Error. PCC: Pearson Correlation Coefficient. (E) and (F) are examples of the 4 pear images with the highest standard deviations from the three technician ratings.
