# Supplementary material for: Rating pome fruit quality traits using deep learning and image processing
Source: Plant Direct. 2024 Oct 8;8(10):e70005. doi: 10.1002/pld3.70005 (PMC11461139; doi:10.1002/pld3.70005)
Supplement: Supplementary file 3 — Figure S1. The fruit detection module creates a segmentation mask (colored overlay with solid outline), a bound box (correspondingly colored rectangle with dotted outline), and a confidence score (number shown on the up‐left corner of the bound box) for each detected instance. These data will be used for the segmentation of the instance. Figure S2. Examples of fruits detected with the initial model (A) and the extended model (B). One piece of cross‐section was missed in the initial model (indicated with a black arrow in (A)), but is detected with high confidence with the extended model. Although the same false positive region (indicated with yellow stars) was detected in both models, the confidence score is lower in the extended model (0.990) than that in the initial model (0.996). Figure S3. Superficial scald rating workflow. Figure S4. Histogram along the a* channel in the CIELAB space of an apple image for demonstrating the scald thresholding method. (A) shows the 0–255 range of the a*channel. Pixel bins increment by 1 from 0 to a maximum of 255. The region in the black box in (A) is expanded as (B). The lower limit is defined as the pixel bin with the lowest pixel values in the image, whereas the upper limit is the pixel bin with the highest pixel value. The maximum pixel bin is defined as the bin with the most pixels. The X‐axis shows the range of pixel bins, y‐axis is the relative abundance of pixels in each bin. Figure S5. Starch rating workflow. Figure S6. Starch pattern indices (SPI) are commonly used for starch content assessment. From top to bottom, the SPIs are the generic starch index card developed by Cornell University (as known as the Cornell Chart); the generic starch index card developed by ENZA Fruit; the starch index card designed for ‘Jonagold’ by O. L. Lau and R. Y. Yastremski; and the ‘Granny Smith’ starch scale developed by UC. Davis. Figure S7. Pear background color rating workflow. Figure S8. Example of the calibration step of the pear blus [file PLD3-8-e70005-s003.pdf]

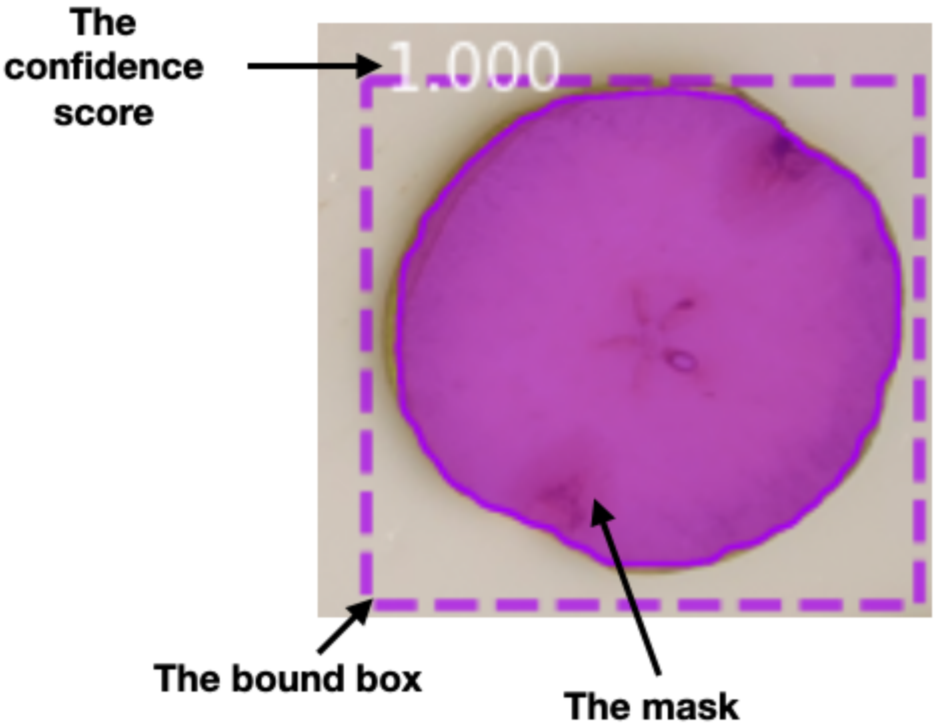

Supplemental Figure 1. The fruit detection module creates a segmentation mask (colored overlay with solid outline), a bound box (correspondingly colored rectangle with dotted outline), and a confidence score (number shown on the up-left corner of the bound box) for each detected instance. These data will be used for segmentation of the instance.

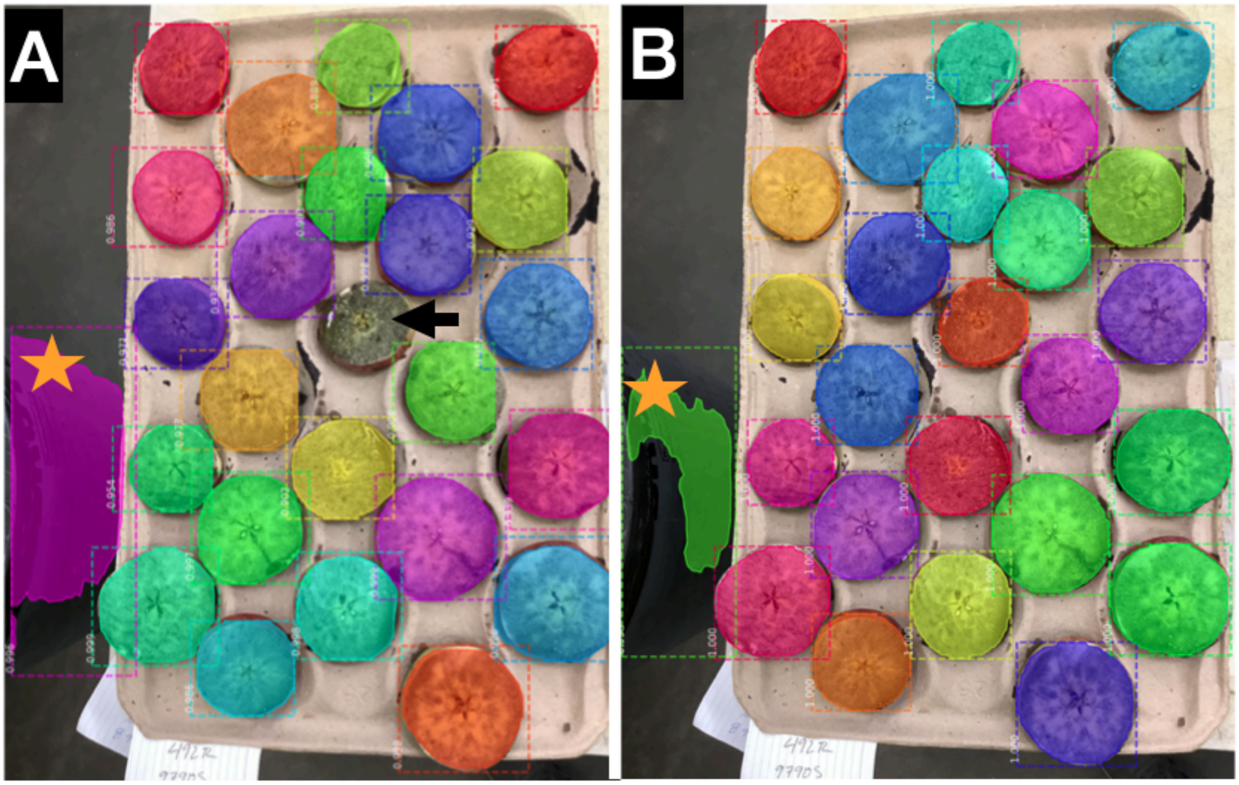

Supplemental Figure 2. Examples of fruits detected with the initial model **(A)** and the extended model **(B).** One piece of cross-section was missed in the initial model (indicated with a black arrow in **(A)**), but is detected with high confidence with the extended model. Although the same false positive region (indicated with yellow stars) was detected in both models, the confidence score is lower in the extended model (0.990) than that in the initial model (0.996)

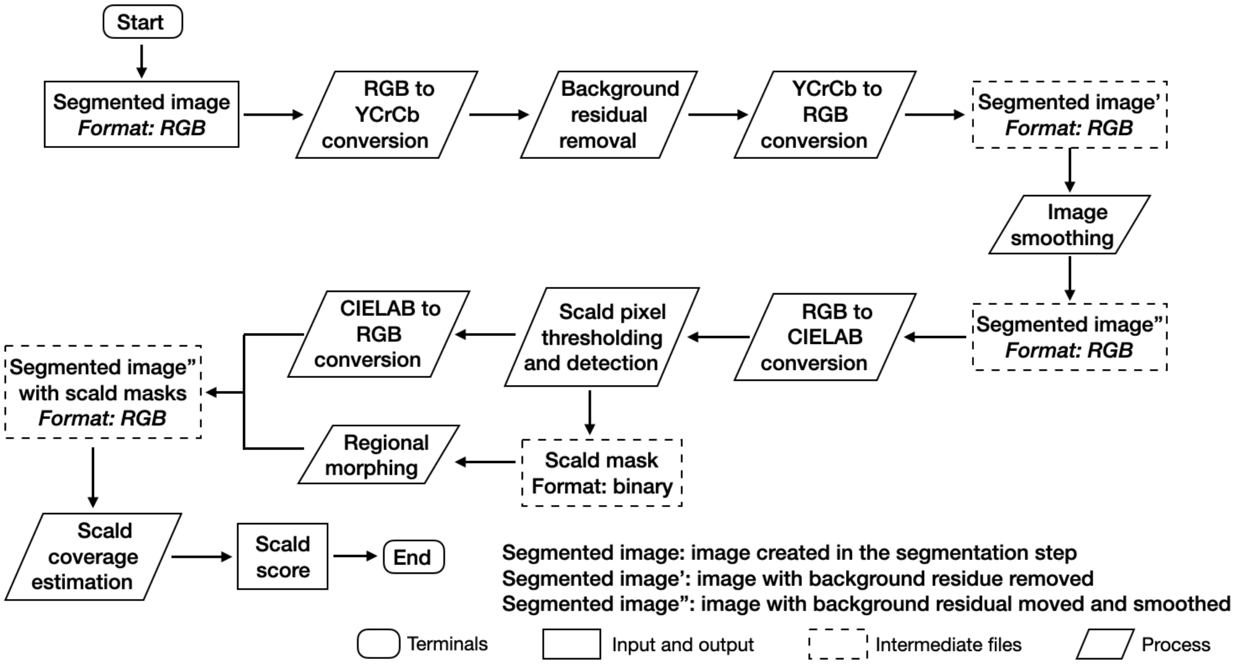

Supplemental Figure 3. Superficial scald rating workflow

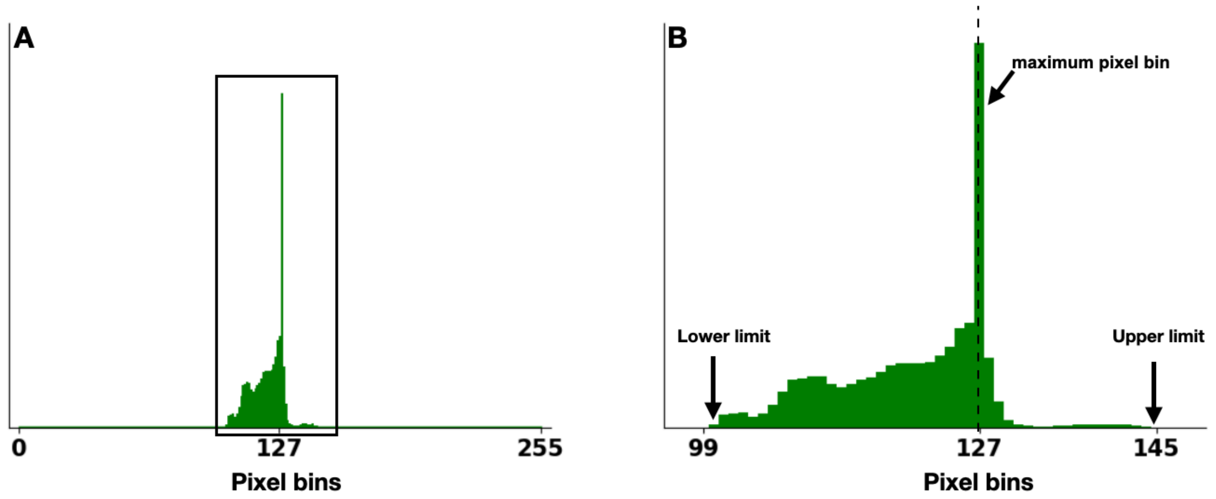

Supplemental Figure 4. Histogram along the a* channel in the CIELAB space of an apple image for demonstrating the scald thresholding method. (A) shows the 0-255 range of the a* channel. Pixel bins increment by 1 from 0 to a maximum of 255. The region in the black box in (A) is expanded as (B). The lower limit is defined as the pixel bin with the lowest pixel values in the image, whereas the upper limit is the pixel bin with the highest pixel value. The maximum pixel bin is defined as the bin with the most pixels. X-axis shows the range of pixel bins, y-axis is the relative abundance of pixels in each bin.

The definitions of histogram, range, and pixel bin are adopted from:
https://docs.opencv.org/3.4/d8/dbc/tutorial_histogram_calculation.html

Below shows the methods for threshold determination:

Apple pixel range = upper limit - lower limit

Threshold = maximum histogram pixel bin - 1/3 apple pixel range

For the example shown in here, the threshold is calculated as below:

Threshold = 127 - 1/3 (145-99) = 111.67

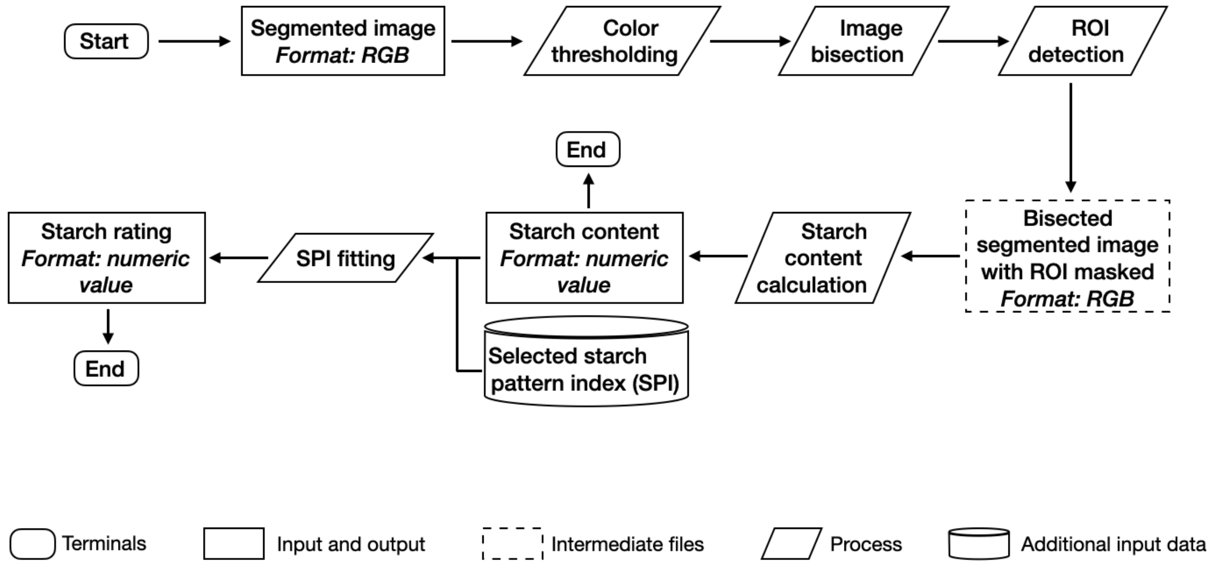

Supplemental Figure 5. Starch rating workflow

Cornell
Starch-Iodine Index

McIntosh should be picked for CA storage as the average starch staining in flesh tissue changes from 60 and 40 percent; i.e., at starch-iodine index 5 and 6. Empire are usually at the best stage of fruit development for CA harvest when the average starch-iodine index for several blocks is 4.5 to 5.5. The harvest windows for CA Delicious and CA Idared usually occur when the starch-iodine indexes are between 2.8 and 3.5.

Starch-iodine indexes for the harvest windows of other varieties have not yet been determined. If you do not have these four varieties to estimate the harvest windows for intervening varieties, the usefulness of the starch-iodine index is limited to comparisons of the current season with previous seasons.

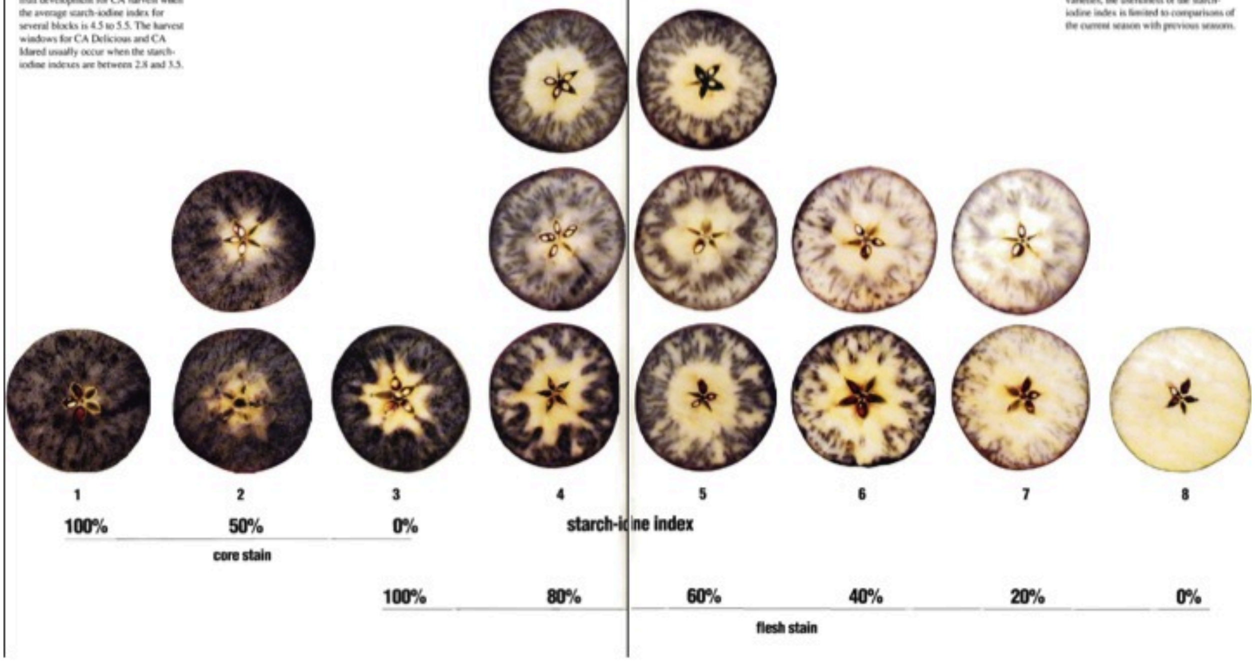

Blanpied, G.D. and S.J. Silsby. 1992, Predicting Harvest Date Windows for Apples. Cornell Cooperative Extension. Informational Bulletin 221.

PIPFRUIT NEW ZEALAND

STARCH PATTERN INDEX FOR APPLES

ENZAFRUIT

ENZAFRUIT is the trading name of ENZA Limited

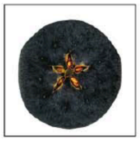

- This chart accounts for variations between apple varieties and is suitable for assessing all starch patterns after iodine solution staining.
- Use a 20 fruit sample for meaningful results.
- Score fruit based on unstained area, not intensity of staining.
- Refer to ENZAFRUIT Maturity Procedures Manual for further measurement procedures.

Description of Starch Pattern

0 full starch/stain black
1 area within the core line clear/unstained
2 clear to vascular bundles
3 25% fresh clear/unstained, area within cortex patchy white
4 33% fresh clear/unstained, area within cortex patchy white
5 66% fresh clear/unstained, area within cortex patchy white
6 starch/stain just under the skin, odd patch of black stain within cortex
7 no starch/100% clear

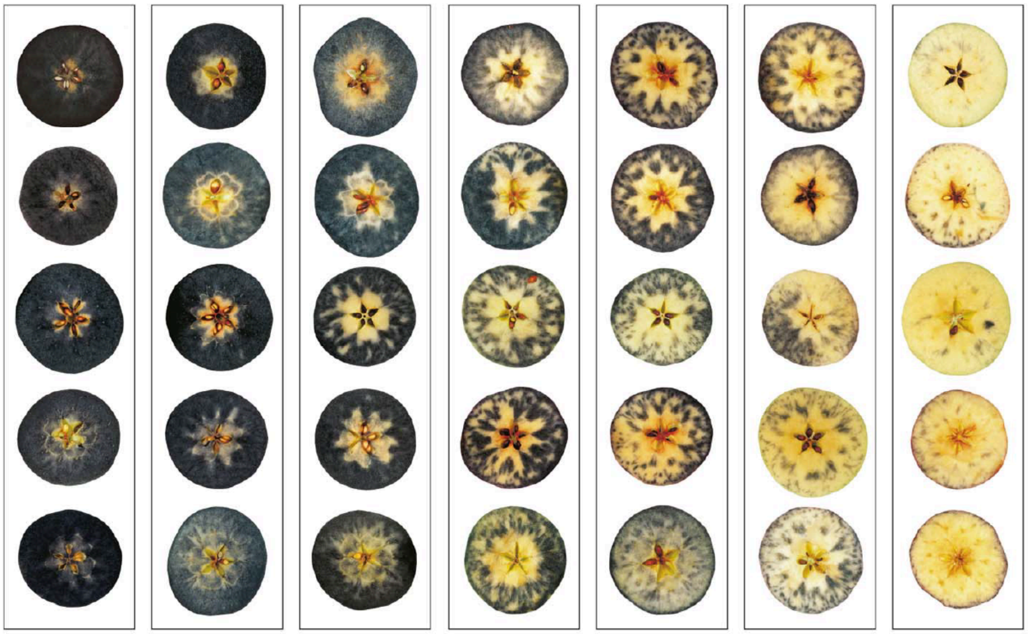

0 1 2 3 4 5 6 7

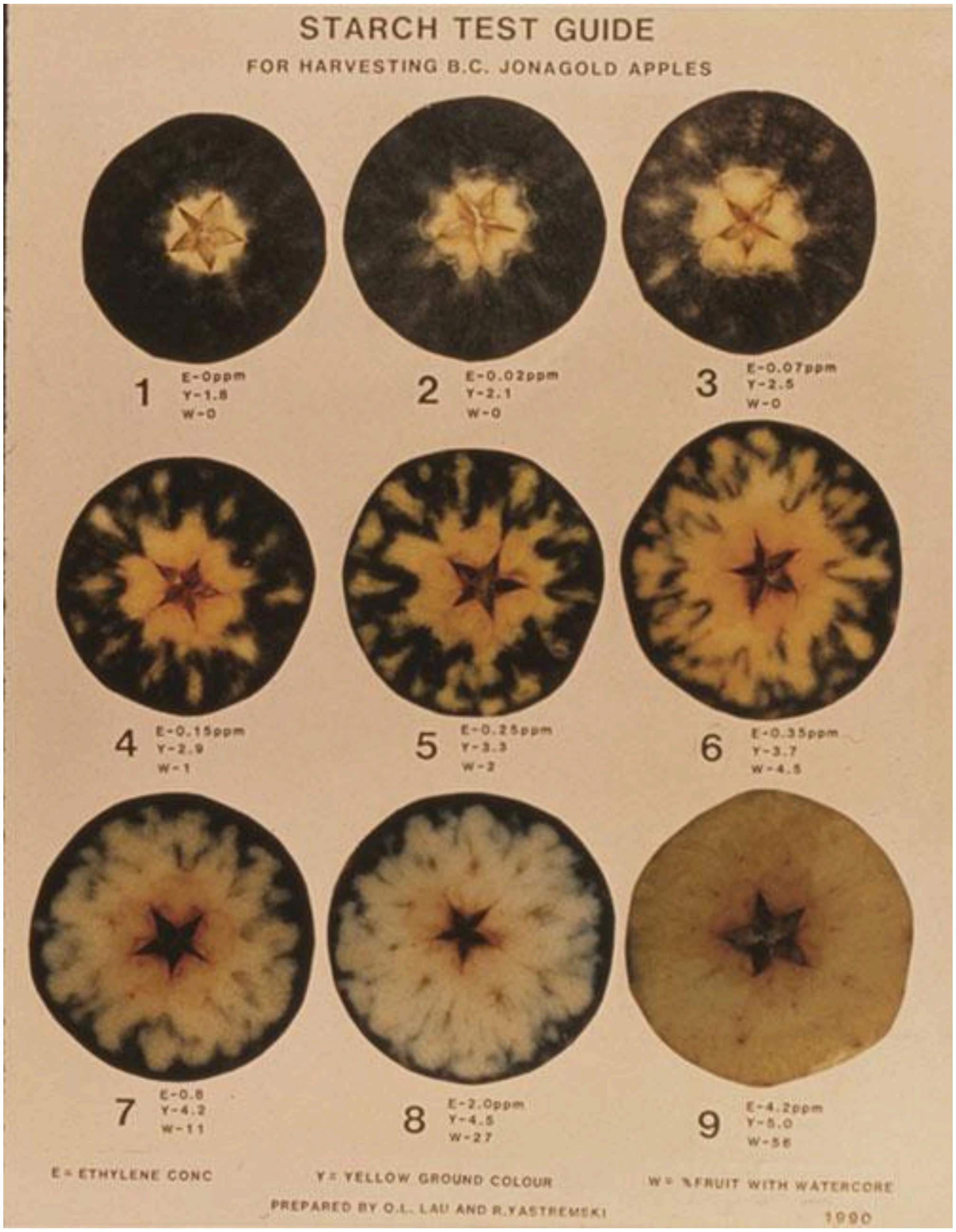
STARCH TEST GUIDE
FOR HARVESTING B.C. JONAGOLD APPLES
1
E-0ppm
Y-1.8
W-0
2
E-0.02ppm
Y-2.1
W-0
3
E-0.07ppm
Y-2.5
W-0
4
E-0.15ppm
Y-2.9
W-1
5
E-0.25ppm
Y-3.3
W-2
6
E-0.35ppm
Y-3.7
W-4.5
7
E-0.8
Y-4.2
W-11
8
E-2.0ppm
Y-4.5
W-27
9
E-4.2ppm
Y-5.0
W-56
E= ETHYLENE CONC
Y= YELLOW GROUND COLOUR
W= %FRUIT WITH WATERCORE
PREPARED BY O.L. LAU AND R.YASTREMSKI
1990

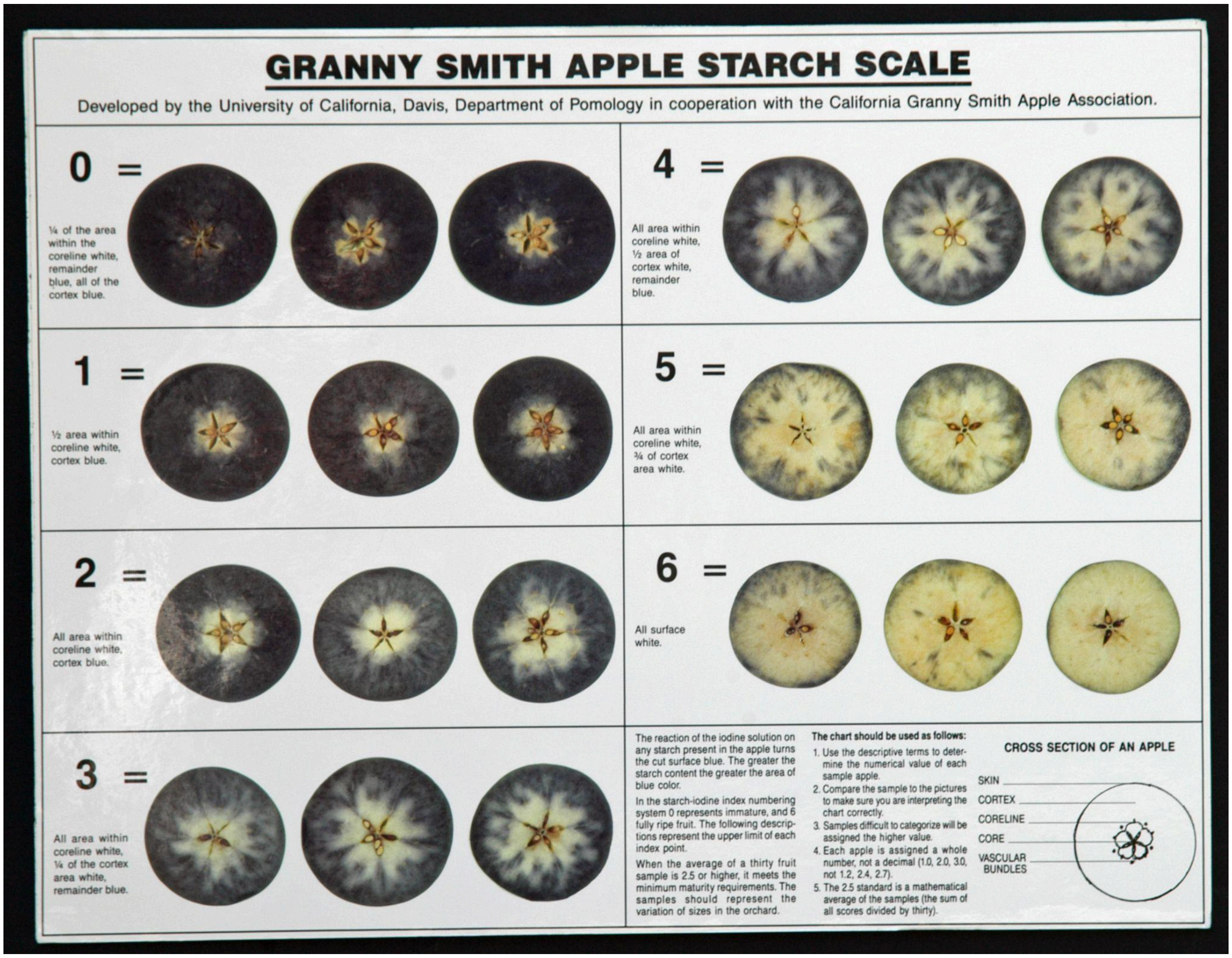

Supplemental Figure 6. Starch pattern indices (SPI) commonly used for starch content assessment. From top to bottom, the SPIs are: The generic starch index card developed by Cornell University (as known as the Cornell Chart); The generic starch index card developed by ENZA Fruit; The starch index card designed for ‘Jonagold’ by O. L. Lau and R. Y. Yastremski; and the ‘Granny Smith’ starch scale developed by UC. Davis.

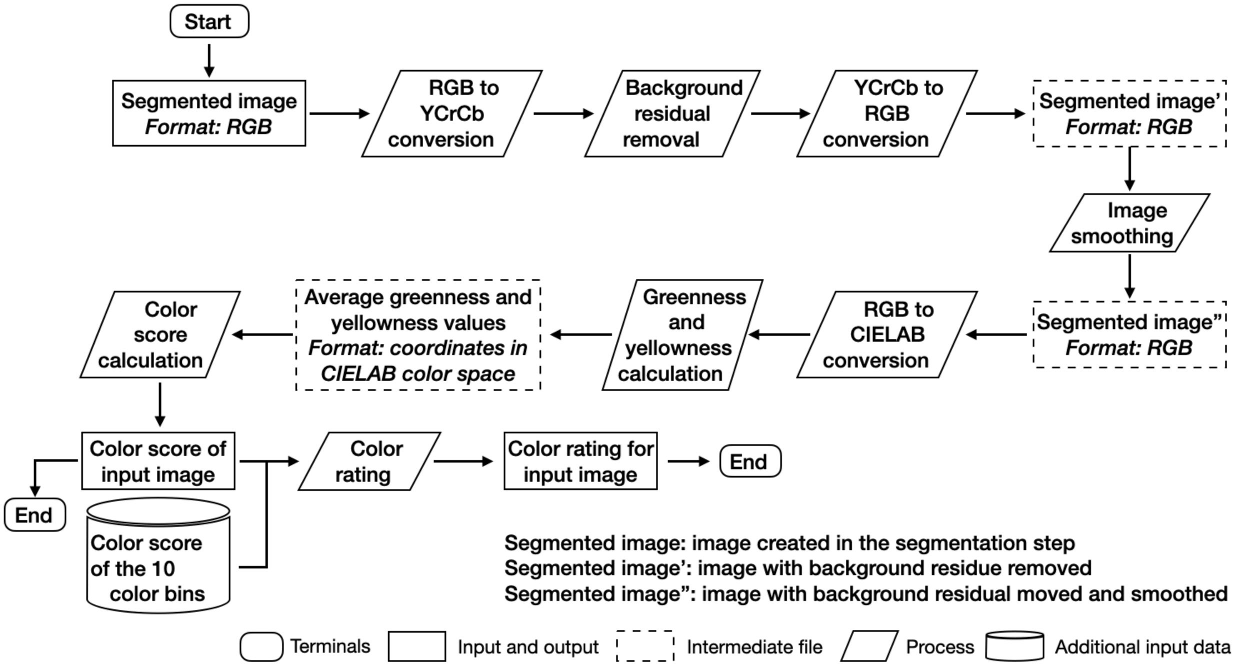

Supplemental Figure 7. Pear background color rating workflow

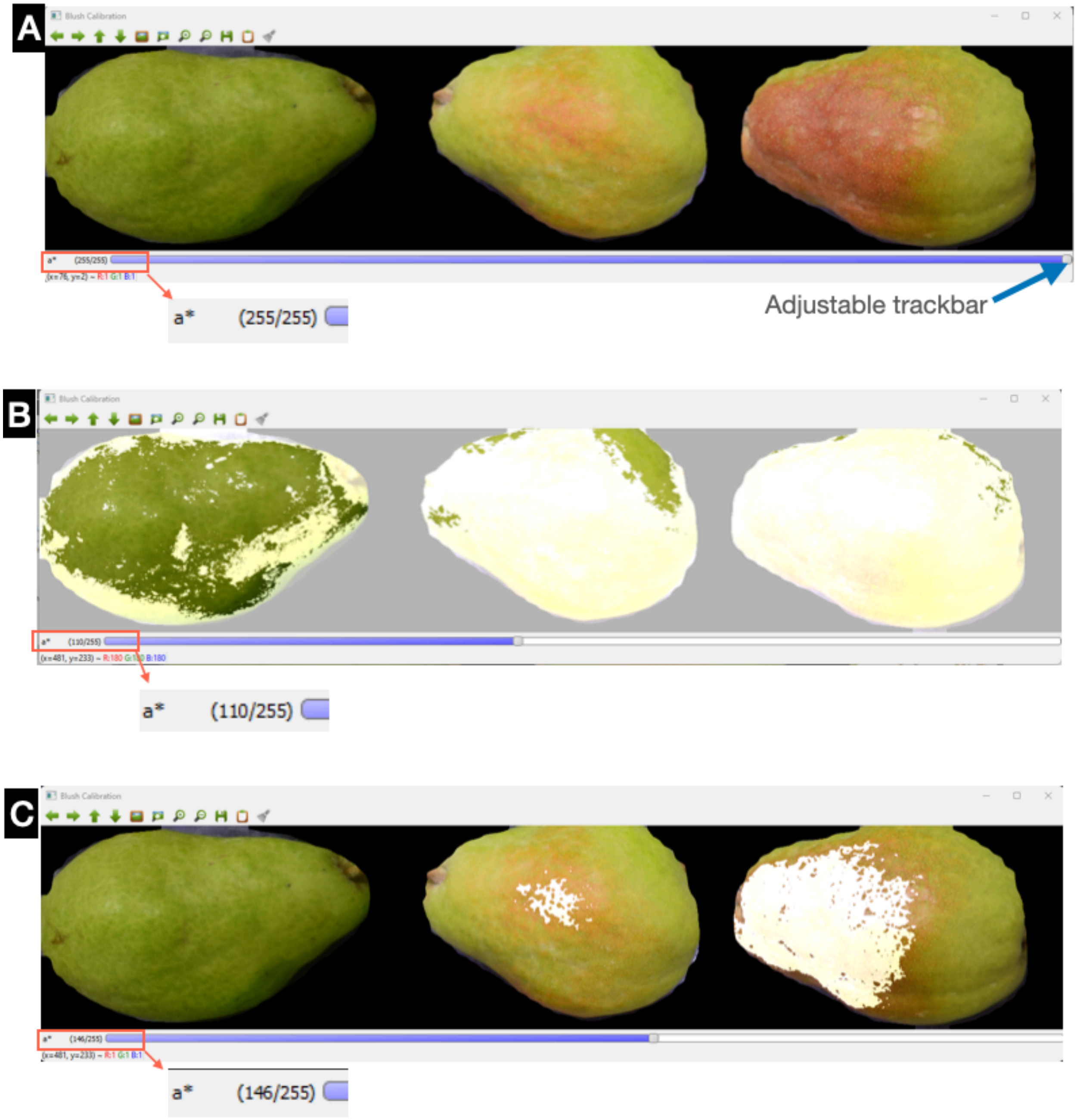

Supplemental Figure 8. Example of the calibration step of the pear blush module visualization in an OpenCV window. **(A)** shows the three selected pear images representing no blush, light-colored blush, and intense-colored blush, from left to right. The trackbar, located on the bottom of the interface, can be used to adjust the threshold of the a* channel. The current a* channel reading and the max reading is shown to the left of the trackbar. **(B)** and **(C)** show the masks overlaying the pear images after adjusting the threshold using the trackbar. The mask in **(B)** covers only the blush region, but also green peel regions. The mask in **(C)** fails to cover all the blush regions. An ideal threshold would be between **(B)** and **(C)**.
