# Supplementary material for: Rating pome fruit quality traits using deep learning and image processing
Source: Plant Direct. 2024 Oct 8;8(10):e70005. doi: 10.1002/pld3.70005 (PMC11461139; doi:10.1002/pld3.70005)
Supplement: Supplementary file 4 — Data S1. Supporting Information. [file PLD3-8-e70005-s004.pdf]

Plant Direct
Open Access

Rating Pome Fruit Quality Traits Using Deep Learning and Image Processing

Nhan H. Nguyen[1], Joseph Michaud[2], Rene Mogollon[1,3], Huiting Zhang[1,4], Heidi Hargarten[4], Rachel Leisso[2], Carolina A. Torres[1,3], Loren Honaas[4]*, Stephen Ficklin[1]*

Decision Letter Round 1:

July 6, 2024

Stephen Ficklin
Washington State University
Pullman, Washington

RE: Rating Pome Fruit Quality Traits Using Deep Learning and Image Processing

Dear Dr. Ficklin:

Thank you for submitting to Plant Direct. All required reviews have been returned and we have now finished our evaluation of your manuscript. In light of the reviewers' and editor's comments, further revisions are needed before the paper can be accepted for publication in Plant Direct.

Please view the editors' and reviewers' comments below and use their suggestions as a guide while you work on your revision.

When uploading the revised version of this article, please be sure to include the following:

-A word document that contains your response to the reviewers. You should respond to each reviewer comment and note the changes made to the manuscript. If you do not agree with a reviewer's comment and choose not to make a suggested revision, please explain why. Please try to provide as complete an answer as possible to each reviewer's criticisms.
-A tracked changes document with each change highlighted
- A clean version of the latest version of the manuscript

I had some trouble finding suitable reviewers. I have secured one review that provides sufficient feedback. In the interest of not delaying the decision any further, I'll ask that

you provide a detailed response to this review in a revised version.

To upload your revision, please click the link below.
https://plantdirect.msubmit.net/cgi-bin/main.plex?el=A1Lr5gl4A1old7I7A9ftdYDo6YjmHwexS65pVTSLM4wZ

In order to provide as timely a service as possible, we ask that your revision is resubmitted within three months after receipt of this request. If an extension is needed, please send a request, along with a brief explanation, to the editorial office at plantdirect@wiley.com .

Please note that, in addition to publishing reviewer comments, the author's responses to review comments will also be published alongside the final version of the paper. If you would not like the author's responses to be published, please contact the editorial office at plantdirect@wiley.com .

Thank you very much for giving us an opportunity to review your work. I look forward to receiving the next version.

Sincerely,

Gustavo MacIntosh

Editor, Plant Direct

-- Editor comments:
I had some trouble finding suitable reviewers. I have secured one review that provides sufficient feedback. In the interest of not delaying the decision any further, I'll ask that you provide a detailed response to this review in a revised version.

-- Reviewer comments:
Reviewer #2:

The ms presents a tool for automated rating of apple and pear fruit quality traits, this would be useful to reduce the bias of human-based visual assessment. I provide some

comments in the hope they can be of assistance to further improve the paper.
The reader would expect the analysis of residuals to be conducted in any modelling exercise, to better support any conclusions
In places (e.g., L350) the authors refer to "cultivar-specific characteristics", however it is unclear whether Granny is able to work in more cultivars or not; please specify in the text the cultivar used for training (e.g., L199). Moreover, at L 340, the scald region was segmented over a background "of green apple fruit peel", would this procedure be scalable to non-green cultivars?
Replace non-SI units, e.g. "pounds", check the whole paper
L55-56 "range of symptoms"..."physiological disorders" please provide some examples

L114-117, should this comment be moved under Discussion

L187, please justify the use of 2,000 pixels2 threshold

please note that RMSE is a scale-dependent parameter, hence a measuring unit is expected

Decision Letter Round 2:

September 6, 2024
Stephen Ficklin
Washington State University
Pullman, Washington

MSID: 2024-01453R1
MS TITLE: Rating Pome Fruit Quality Traits Using Deep Learning and Image Processing

Dear Dr. Stephen Ficklin:

I am pleased to inform you that your manuscript "Rating Pome Fruit Quality Traits Using Deep Learning and Image Processing" has been accepted for publication in Plant Direct.

Your article will appear online in the next available issue of Plant Direct. To ensure your article gets published as quickly as possible, please pay attention to the steps detailed below. We have found that most of the delays happen at this stage, especially at the

payment stage, so please respond as quickly as possible when prompted.

License Agreement: Once your article has been accepted it will move to Production and undergo admin and file checking - you may receive an email with any queries we have at this stage. When all required items are received by the publisher and queries resolved, the corresponding author will receive an email from Wiley's Author Services system which will ask them to log on at https://authorservices.wiley.com/bauthor and will present them with the appropriate license for completion. Your article cannot be published until both the signed license agreement and payment of the article fee have been received.

Payment of the Open Access Article Publication Fee: All articles published in Plant Direct are fully open access: immediately and freely available to read, download and share. Plant Direct charges a publication fee to cover the publication costs. The corresponding author for this manuscript should have already received a quote from the payments team (cs-openaccess@wiley.com) with the estimated article publication fee; please email cs-openaccess@wiley.com if this has not been received. The corresponding author should log on to the Wiley Author Services site, where the publication fee can be paid by credit card or an invoice. Pro Forma can also be requested. Payment of the publication charge must be received before the article will be published online.

Proofs: You will have the opportunity to look over your paper once more when you receive the author proofs for your article. The proofs will be with you in approximately two weeks. Please note that, in addition to publishing reviewer comments, the author's responses to review comments will also be published alongside the final version of the paper. If you would not like the author's responses to be published, please contact the editorial office at plantdirect@wiley.com .

Promotion of your article: You can help your research get the attention it deserves! Check out Wiley's free Promotion Guide for best-practice recommendations for promoting your work at www.wileyauthors.com/eeo/guide. And learn more about Wiley Editing Services which offers professional video, design, and writing services to create shareable video abstracts, infographics, conference posters, lay summaries, and research news stories for your research at www.wileyauthors.com/eeo/promotion.

Thank you again for your contribution to Plant Direct. If you have any questions, feel free to contact the editorial office at plantdirect@wiley.com .

Sincerely,

Gustavo MacIntosh

Gustavo MacIntosh

Editor, Plant Direct

Response to Reviewer:

Reviewer #2:

The ms presents a tool for automated rating of apple and pear fruit quality traits, this would be useful to reduce the bias of human-based visual assessment. I provide some comments in the hope they can be of assistance to further improve the paper.
Thank you for your time reviewing our manuscript!

The reader would expect the analysis of residuals to be conducted in any modelling exercise, to better support any conclusions
We have added supplemental figure 9G & H (see below) to document the residuals of Figure 4. This is the only plot where we used a linear model to compare two quantitative measurements (human and Granny) and calculate an RMSE value to indicate performance. All other figures used to show performance have a categorical human rating.

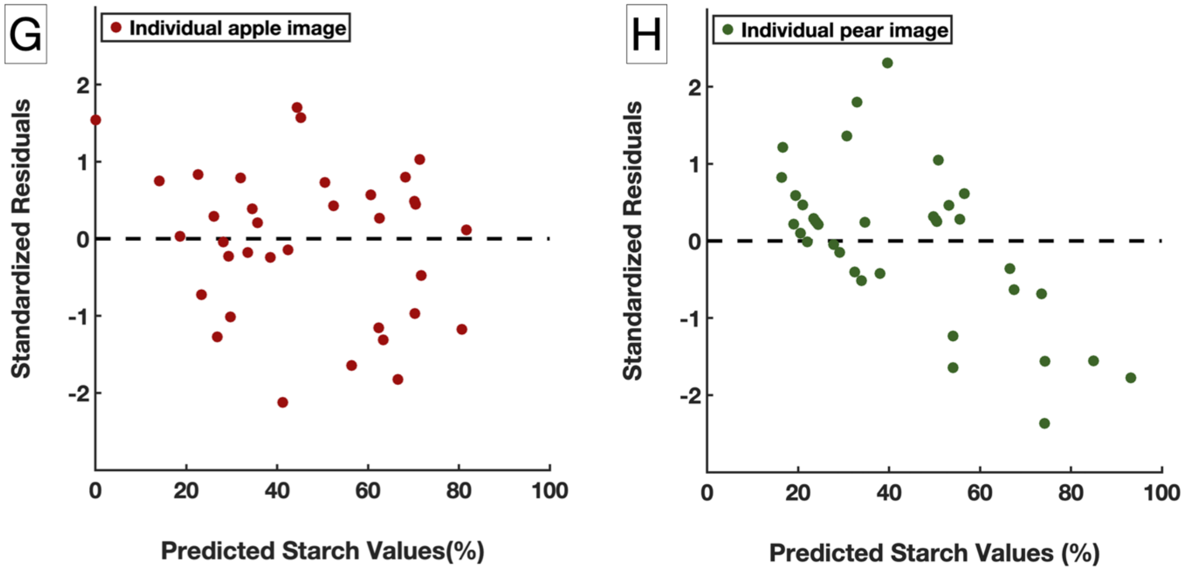

In places (e.g., L350) the authors refer to "cultivar-specific characteristics", however it is unclear whether Granny is able to work in more cultivars or not; please specify in the text the cultivar used for training (e.g., L199). Moreover, at L 340, the scald region was segmented over a background "of green apple fruit peel", would this procedure be scalable to non-green cultivars?
Yes, Granny can work with multiple cultivars for rating starch clearing. The end of that paragraph states this but that was not very clear. We have updated that paragraph to hopefully clarify that Granny will provide as output its high-resolution value, but also approximate values that correspond to various, commonly-used starch rating cards, some of which are cultivar-specific.

Replace non-SI units, e.g. "pounds", check the whole paper
We only found one location (first paragraph of the introduction) where non-SI units were used. That “11.1 billion pounds” value came from the citation we used, but to address the reviewers request, we included the kilogram conversion.

L55-56 "range of symptoms"..."physiological disorders" please provide some examples
We have added examples for symptoms and disorders. The sentence now reads as: “During storage, apples and European pears can display a wide range of physiological disorders such as bitter pit, core flush, browning, superficial scald, and sun scald, affecting fruit marketability. Additionally, other losses in quality such as undesired color can affect final fruit packout from packinghouses”

L114-117, should this comment be moved under Discussion

We see why the reviewer made this suggestion, the grammar and meaning was a bit incorrect and made that statement look like a clarifying statement. It has been corrected and hopefully indicates the purpose for the model selection.

L187, please justify the use of 2,000 pixels2 threshold

For the ImageJ function used for starch clearing ratings, we selected a size of 2000 through trial-and-error and it worked well to exclude small artifacts, and other noise. We have updated the manuscript to make this clear. We text now reads:
"To reduce this noise, identified ROIs smaller than 2,000 pixels^2 are excluded from the ROI measurement. We chose 2000 pixels because it performed well at removing and excluding small artifacts and other noise"

please note that RMSE is a scale-dependent parameter, hence a measuring unit is expected

We have adjusted the caption of Figure 4 to indicate that RMSE is of the percentage of starch to non-starch regions of the apple.

Note to reviewer: In addition to the updates to the manuscript requested by the reviewer we added some very brief clarification at the end of the document about the release version of the software so that it is clear to the reader which version of Granny the paper is for.
